# Translation and Test–Retest of the Spanish Podiatry Health Questionnaire (PHQ-S)

**DOI:** 10.3390/ijerph15102205

**Published:** 2018-10-10

**Authors:** Emmanuel Navarro-Flores, Marta Elena Losa-Iglesias, Ricardo Becerro-de-Bengoa-Vallejo, Daniel López-López, David Rodríguez-Sanz, Patricia Palomo-López, César Calvo-Lobo

**Affiliations:** 1 Faculty of Nursing and Podiatry, Universidad de Valencia, 46010 València, Spain; manu.navarroflores@gmail.com; 2 Faculty of Health Sciences, Universidad Rey Juan Carlos, 28933 Móstoles, Spain; marta.losa@urjc.es; 3 Facultad de Enfermería, Fisioterapia y Podología, Universidad Complutense de Madrid, 28040 Madrid, Spain; ribebeva@ucm.es (R.B.-d.-B.-V.); davidrodriguezsanz@gmail.com (D.R.-S.); 4 Research, Health and Podiatry Unit, Department of Health Sciences, Faculty of Nursing and Podiatry, Universidade da Coruña, 15001 A Coruña, Spain; 5 Faculty of Sports Sciences, European University of Madrid, Villaviciosa de Odón, 28670 Madrid, Spain; 6 University Center of Plasencia, Universidad de Extremadura, 06071 Badajoz, Spain; patibiom@unex.es; 7 Nursing and Physical Therapy Department, Faculty of Health Sciences, Universidad de León, 24071 León, Spain; cecalvo19@hotmail.com

**Keywords:** foot, quality of life, health impact assessment, validation studies

## Abstract

*Background*: The Podiatric Health Questionnaire (PHQ) is a health-related questionnaire consisting of six questions designed for measuring foot health status. To date, the PHQ has only been validated in the English-language version. Thus, this study aimed to perform the Spanish translation and test–retest procedures of the PHQ (PHQ-S). *Method*: The forward/backward translation and test–retest reliability methods were applied from English to Spanish languages. Regarding the total score for each domain, internal consistency and reliability were determined by the Cronbach α and intraclass correlation coefficient (ICC) with a confidence interval (CI) of 95%. *Results*: High internal consistency was shown for the six domains: (1) walking with a Cronbach α of 0.97; (2) hygiene and nail care with 0.93 and 093, respectively; (3) foot pain with 0.91; (4) worry and concern domain with 0.904; (5) quality of life with 0.87; and (6) the self-perception of how their feet are feeling measured by a visual analogic scale with 0.92. Excellent test–retest reliability (ICC = 0.99 (95% CI = 0.96–0.98)) was shown for the total score. *Conclusions*: The PHQ-S was shown to be a valid and reliable tool for an acceptable use in the Spanish population.

## 1. Introduction

Worldwide, clinimetric analyses for tools such as the Foot Health Status Questionnaire (FHSQ), the Foot Function Index (FFI) and the Manchester Foot Pain and Disability Index (MFPDI) were carried out in order to validate and translate these tools for assessing the quality of life (QoL) related to foot health [1,2,3,4]. Foot conditions may be present in approximately 17% to 42% of the adult population [5]. Indeed, up to 8% of musculoskeletal pain consultations carried out by general practitioners were associated with foot and ankle alterations [6]. This prevalence may increase in older adults who present foot pain with specific alterations such as hyperkeratosis lesions, hallux valgus, plantar warts, fungus, nail changes, sprains, and lesser toe deformities [7,8,9,10,11,12], all of which are associated with higher disability [13]. In addition, an impaired health-related quality of life (QoL) and an increased fall risk may be related to these foot disorders [14,15].

The Podiatric Health Questionnaire (PHQ) may be defined as a self-reported health questionnaire in order to evaluate foot problems and their impact in the QoL related to foot health. It consists of six domains such as walking, foot pain, hygiene, nail care, worry and concern, health-related QoL, and self-perception of how their feet are feeling by a Visual analogic scale (VAS). The PHQ was initially developed in the United Kingdom (UK) with an appropriated concurrent validity [16]. This questionnaire has been previously administered to podiatric patient populations [17]. A good level of concordance was reported between the PHQ, EuroQoL-5D (EQ-5D), and Podiatry Objective Clinical Score (POCS) [16,18]. Thus, the PHQ reflects the patients’ perceptions of their foot health status and QoL in addition to accurately evaluating post-treatment efficacy and determining foot health within populations [16].

Regarding the PHQ domains, six underlying factors may be considered in order to evaluate different QoL dimensions related to foot health status [16]. Translation and test–retest procedures should be performed according to the guidelines in order to maintain cross-cultural measurement properties [3,19,20,21]. Currently, the PHQ has not been adapted or validated in Spanish [16,17,22]. Indeed, the PHQ may be easier and simpler for administration and measurement as well as provide new specific items, such as walking, hygiene, nail care, worry and concern or the self-perception of how their feet are feeling measured by a VAS [16,17], compared to prior clinimetric analyses of tools for assessing the QoL related to foot health, such as the FHSQ, FFI and MFPDI [1,2,3,4]. Thus, this study aimed to perform the Spanish translation and test–retest procedures of the PHQ (PHQ-S).

## 2. Methods

### 2.1. Participants and Public Involvement

Fifty-two participants (33 females and 19 males) with mean ± SD (lower–upper limits of the 95% confidence interval) age, weight, height, and body mass index (BMI) of 45.78 ± 20.02 (40.21–51.36) years old, 66.88 ± 10.96 (63.83–69.93) kg, 166 ± 0.09 (163–168) cm, and 24.29 ± 3.82 (23.22–25.35) kg/cm^2^, respectively, were recruited from the Podiatry and Physiotherapy Clinical Center at University of Extremadura in the city of Plasencia, Cáceres (Spain). Inclusion criteria comprised participants who presented foot pain for at least three months. Exclusion criteria comprised psychiatric or cognitive disorders, neuropathy, systemic disorders or participants under pain killing medications, being registered in the medical record, as well as refusal to participate or not sign the informed consent form and the inability to follow the instructions necessary to participate in the present study. Participants were recruited concurrently through direct approach and voluntarily required to fill out the tool as part of their normal consulting appointment.

### 2.2. Study Design

A translation and test–retest study was carried out between November 2017 and February 2018 according to the Patient-Reported Outcome Measures (PROMs)—Principles of Good Practice statement and checklist [1,23]. The translation and test–retest procedures were developed using the PHQ as a foot health-related QoL tool in order to determine its clinimetric properties [16].

### 2.3. Ethical Statements

Ethical approval was obtained by the Committee from the Bioethics and Biosafety Committee at the University of Extremadura (Spain). Furthermore, informed consent was obtained from all subjects. The Helsinki Declaration, the Organic Law of Protection Data (reference 15/1999; i.e., a Spanish law for protection of the patients´ information) and human experimentation ethical standards were respected.

### 2.4. Translation Procedure

The forward/backward translation protocol was used for the translation, cross-cultural adaptation, and validation procedure from the UK version to the Spanish version [2,3,19,20,21]. According to prior recommendations, the translation procedure was conducted following international guidelines [19,23].

Firstly, the author of the original PHQ version was contacted and asked to perform this translation [16]. Secondly, forward translation was performed by two independent bilingual Spanish translators (forward). Thirdly, these translations were separately reconciled by each translator (backward). Fourthly, the reconciled forward-translated version from the PHQ-S was translated into Spanish by seven authors (backward). Fifthly, the translated version was compared with the original version to verify the conceptual equivalence of the translation, discrepancy, or unclear terms. Sixthly, harmonization occurred via an expert panel formed by seven authors, six podiatrists and one physiotherapist in order to agree about the translation. Seventhly, cognitive interviews were held to the physiotherapy and podiatry centers in order to provide validity and avoid potential errors [23]. Finally, this questionnaire was composed using Likert scales, which improved its administration, and presented adequate psychometric properties [2,16]. The verification and then subsequent expert panel were carried out by the same group who provided the backward translation.

### 2.5. Statistical Analysis

Sociodemographic characteristics (age, sex, weight, height and BMI) and foot conditions were described. Regarding an ICC of 0.40 and a 95% confidence interval (CI) for a two-tailed test, an error α of 0.05, and a desired power of 80% (error β = 20%), a final sample size of 52 participants was calculated. The considered sample was heterogeneous in order to test this questionnaire for various foot conditions [2].

Sociodemographic data, PHQ total score and each domain’s scores (walking and getting about, foot pain, hygiene, nail care, worry and concern, health-related QoL, and the self-perception of how their feet are feeling measured by a VAS) were collected and described as mean ± standard deviation (SD) completed with the lower–upper limits of the 95% confidence interval for test and retest values [16]. Pain was measured separately on a simple VAS; patients were asked to place a mark on a 15 cm line to represent the severity of pain experienced in the past week using a scale from 0 (no pain) to 10 (worst pain). In our study, the VAS was modified by specifying “pain in your foot”.

All variables were tested for normality distribution by means of the Kolmogorov–Smirnov test, and data were considered normally distributed if *p* > 0.05. The total data and all domains studied during the test and retest showed a non-normal distribution (*p* < 0.05), so the distribution was analyzed using the non-parametric paired Wilcoxon signed-rank test in order to test systematic differences between the test and retest. Regarding total score and each domain score, internal consistency and reliability were analyzed using the Cronbach α (α). This parameter was used to summarize the internal correlations of all items on a scale. For clarifying, a higher α coefficient (which ranged from 0.0 to 1.0) was considered more consistent for the scale with a greater likelihood to reflect an underlying single variable on the questionnaire. We examined correlations of all items with the overall score and also whether Cronbach’s α was improved by removal of any item (nevertheless, any item was removed considering the analyzed data).

Reproducibility (test–retest reliability) was assessed by asking 52 patients in the test stage to complete and return a second questionnaire (retest) seven days after the first. The data were examined by the Intraclass Correlation Coefficient (ICC) with a 95% CI. A 2-way random effects model (2.1), single measures, absolute agreement, and ICC were analyzed to express concordance between the test and retest. To interpret ICC values, we used benchmarks as proposed by Landis and Koch [24] with <0.20 as slight agreement, 0.21 to 0.40 as fair, 0.41 to 0.60 as moderate, 0.61 to 0.80 as substantial, and >0.81 as almost perfect.

Construct validity was examined using the Spearman correlation coefficient (according to non-normal data distribution) between the total score of the questionnaire domain measurements obtained at the same assessment both at test and after seven days at the retest.

The use of coefficient of variation (CV) values has been the most common approach used previously for examining variability between tests, and in the current study, a %CV for method error was calculated as follows: CV = 100 × (2 × (SDd /√2)/(X1 + X2) 19. SDd represents the standard deviation of the differences between the two tests, and X1 and X2 represent the two tests’ means. In addition, standard errors of measurement (SEM) were calculated to measure the range of error of each gait parameter. SEM is a quantitative expression of the range of error that can occur whenever the same participant repeats certain tests. In addition, SEM values were calculated from the ICCs and SDs for each session using the higher of the 2 SD measurements to determine the range of error between sessions. SEM was calculated according to the formula SEM = SD × sqrt (1 − ICC). Similarly, and for convenience of interpretation, the percent error of the SEM (SEM%) was calculated as the SEM divided by the mean per 100. The resulting value provided an estimate of the inherent error or variability normalized to the mean. SEM% = SEM/mean × 100 % 20. In addition, in order to determine the smallest amount of change that is real and beyond the bound of measurement error, minimum detectable changes (MDCs) were calculated at a CI of 95%. MDC values, which reflect the magnitude of change necessary to provide confidence that a change was not the result of random variation or measurement error, were calculated as follows 21: MDC = √2 × 1.96 × SEM.

In addition, Bland and Altman graphs were obtained to assess agreement and heteroscedasticity [25].

A *p*-value < 0.05 with a confidence interval of 95% was considered statistically significant for all tests (SPSS for Windows, version 20.0; SPSS Inc., Chicago, IL, USA).

**Figure 1:**
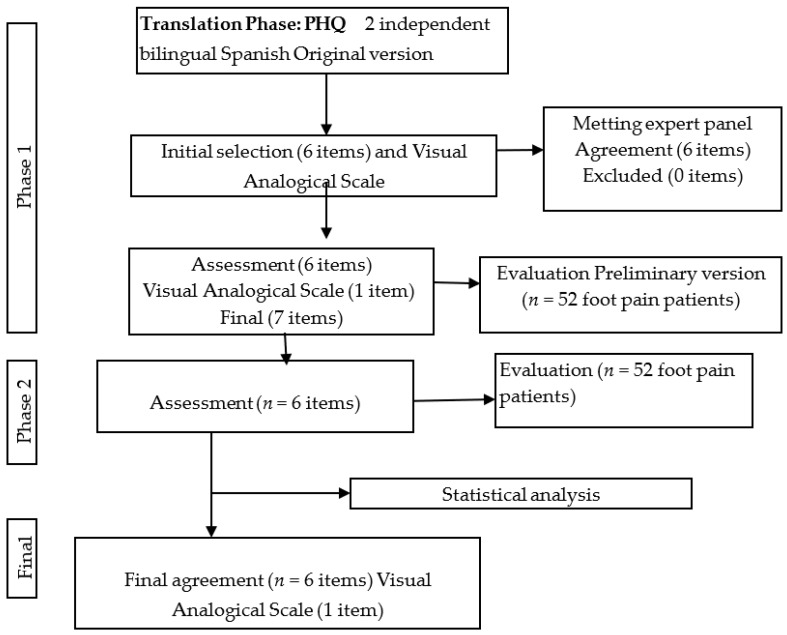
Flow diagram through the research course.

Figure 1 shows the flow diagram through the study course. Test–retest was performed by 52 subjects (including 33 females and 19 males) with age, weight, height, and BMI expressed as mean ± SD (95% CI lower–upper limits); results yielded 45.78 ± 20.02 (40.21–51.36) years, 66.88 ± 10.96 (63.83–69.93) kg, 1.66 ± 0.09 (1.63–1.68) m, and 24.29 ± 3.82 (23.22–25.35) kg/cm^2^, respectively. All variables showed a non-normal distribution (*p* < 0.05).

### 2.6. Translation

The following translations were carried out with only minor discrepancies, and good agreement was observed between the two versions (Table 1). The backward translations between PHQ and PHQ-S were similar for most of the items. Cognitive interviews showed good PHQ-S understanding and comprehension by the participants.

### 2.7. Reliability and Reproducibility

Internal consistency, reproducibility (test–retest reliability), systematic differences and Cronbach α of the PHQ-S questionnaire categorized by questions and domains from the test, retest and VAS are shown in Table 2.

High internal consistency was shown for the six domains, including walking, hygiene, nail care, foot pain, worry and concern, and QoL, and were >0.75 at the test and >0.76 at retest.

Internal consistency by total Cronbach’s α for the study questionnaire was 0.814 at the test assessment (*n* = 52) and 0.82 at retest (*n* = 52). All of the items at test and retest correlated with the total score at >0.5 (Table 2).

Reproducibility showed excellent test–retest reliability for the total score, and each domain, including walking, hygiene, nail care, foot pain, worry and concern, and QoL with ICC were >0.86. The VAS scale showed excellent reproducibility with an ICC of 0.86.

There were no significant systematic differences in any domain and total score between test and retest scores (*p* > 0.05), and VAS did not show differences either between test and retest scores (*p* = 0.834).

Concerning construct validity, the total questionnaire correlated well with the VAS scores at both test and retest at the seven-day follow-up (Table 2), showing an inverse correlation at both test and retest with values of –0.50 and –0.39, respectively (*p* < 0.001).

**Figure 2:**
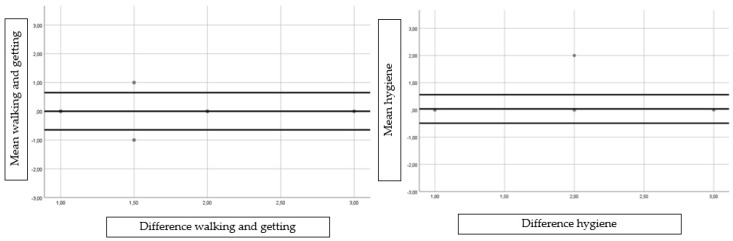

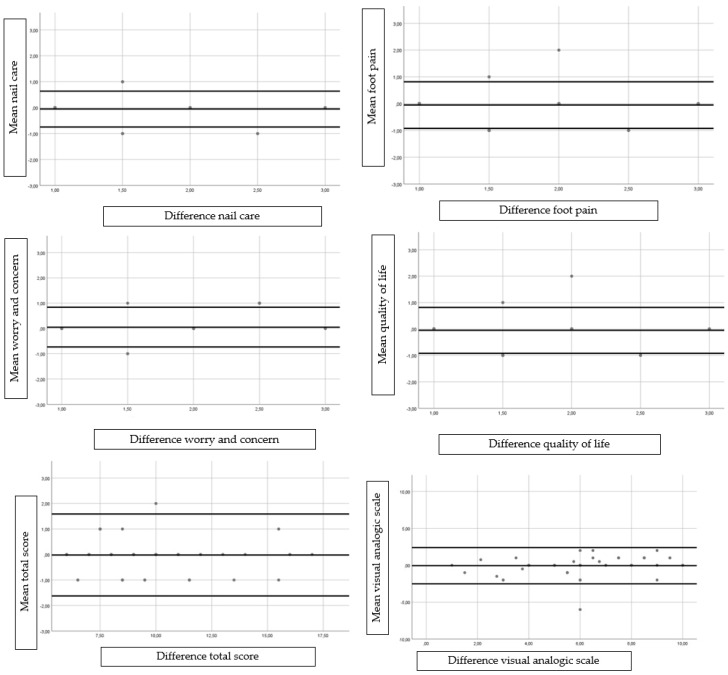
Bland–Altman plot showing the agreement between test and retest for the individual subscales and the total score.

The calculated between-test variabilities (%CV) for each domain and VAS are shown in Table 2, and ranged from 0.12% to 2.68% with very low variability. The MDC values for each domain and VAS, shown in Table 2, ranged from 0.00 to 0.09. For each domain and VAS shown in Table 2, the SEM and SEM% values ranged from 0.00 to 0.03 and from 0.00 to 0.96, respectively. Bland and Altman graph visual distributions did not show any significant or clinically relevant differences between test and retest (Figure 2).

## 3. Discussion

Regarding international recommended guidelines [19,23], the PHQ-S may be used as a valid questionnaire in the Spanish population for measuring the self-reported health impact of foot problems such as walking, foot pain, hygiene, nail care, worry and concern, health-related QoL, and VAS. The original PHQ showed an adequate concurrent validity [16,17].

Previously, various foot health-related questionnaires, Spanish cross-cultural adaptations, and validations were performed with similar results [3,4]. The Spanish version of the PHQ, the PHQ-S, has proven to be a valid and reliable tool showing very good internal consistency (from 0.870 to 0.932) for assessing pain, hygiene, walking, nail care, foot pain, worry and concern, health-related QoL, and self-perception of how their feet are feeling [4]. The general results of the questionnaire showed that the patients were able to score their foot pain degree, which constitutes an encouraging finding inasmuch as the foot self-examination is one of the critical elements related to QoL. These data agreed with those reported by the FHSQ, FFI and MFPDI in order to assess the foot health-related QoL [1,2,3,4], but the administration and measurement of the PHQ-S appear to be easier and simpler than the other tools, because the number of items for each domain and the total questionnaire seem to be lower than the other questionnaires. Nevertheless, future studies should investigate the patients´ preference to complete these questionnaires.

Finally, we should consider some limitations considering this study. Firstly, we deliberately included only people with nonspecific pathologies and only with foot problems; therefore, the questionnaire should be evaluated with the inclusion of other populations with specific pathologies for future studies. Consequently, in the daily clinical practices in Spain, it may only be recommended for patients with general foot problems without indications for specific pathologies. Secondly, the PHQ-S was carried out from podiatry and physiotherapy clinics in which university students perform their practices, while the original PHQ was developed from podiatry services in clinic and domiciliary locations across four National Health Service Trusts in Yorkshire and Humberside, UK [16]. Future studies with different populations should be carried out to test the tool in different contexts of foot care and cultural diversity. For example, we think the foot health-related QoL context in Spain may differ from the UK, since nowadays podiatry is not included in the public health service. Thirdly, different age distributions (such as children) were not included in this Spanish version validation, while different scales were considered in this age range (such as in the Oxford Ankle Foot Questionnaire) which was validated in children from five to 16 years old [26]. Thus, we can only recommend this questionnaire for the studied age distribution, while future studies should include all age ranges. Finally, the authors were also clearly not blinded to the intent of the backward translation and were assumedly familiar with the PHQ itself, which can provide significant bias.

## 4. Conclusions

The PHQ-S was shown to be a valid and reliable questionnaire with an acceptable use in the Spanish population, and may be used for total or each domain scores, such as walking, foot pain, hygiene, nail care, worry and concern, QoL, and self-perception of how their feet are feeling.

## Figures and Tables

**Table 1:** Translations between the two versions of the Podiatric Health Questionnaire: PHQ and PHQ-S.

Questions of Each Domain	Cuestiones de Cada Dominio
Walking/getting about *How much do your feet affect your walking about?* I have no problems in walking about I have some problems in walking about I have severe problems walking about	Para caminar o moverse *¿Cuánto te influyen tus pies para caminar o moverte?* No tengo problemas con los pies para caminar Tengo algunos problemas con los pies para caminar Tengo problemas graves con los pies para caminar
Hygiene *How much of a problem is it for you to wash and dry your feet?* I have no problem washing or drying my feet I have some problems washing and drying my feet I cannot wash or dry my feet	Higiene de los pies *¿Tienes para lavar y secar los pies?* No tengo problemas para lavarme o secarme los pies Tengo algunos problemas para lavarme y secarme los pies No puedo lavarme ni secarme los pies
Nail care *How much of a problem is it for you to cut your own toe nails?* I have no problems cutting my own toe nails I have some problems cutting my own toe nails I cannot cut my own toe nails	Corte de uñas de los pies *¿Qué dificultades tienes para cortar sus propias uñas?* No tengo dificultades para cortarme las uñas de los pies Tengo algunos dificultades para cortarme las uñas de los pies No puedo cortarme las uñas de los pies
Foot pain *Do your feet cause you pain or discomfort?* My feet cause me no pain or discomfort My feet cause me some pain or discomfort My feet cause me severe pain or discomfort	Dolor de pies *¿Tus pies te causan dolor o molestias?* Mis pies no me causan dolor ni molestia Mis pies me causan algo de dolor o molestias Mis pies me causan dolor o molestias severas
Worry/concern *How much are you concerned about the condition of your feet?* I am not concerned about the condition of my feet I am somewhat concerned about the condition of my feet I am greatly concerned about the condition of my feet	Preocupación *¿Cuánto te preocupa el estado de tus pies?* No estoy preocupado por el estado de mis pies Estoy algo preocupado por el estado de mis pies Estoy muy preocupado por el estado de mis pies
Quality of life *Do your foot problems affect the quality of your life?* My foot problems do not affect the quality of my life My foot problems have some effect on the quality of my life My foot problems have a severe effect on the quality of my life	Calidad de vida *¿Tus problemas con los pies, afectan la calidad de su vida?* Mis problemas en los pies no afectan la calidad de mi vida Mis problemas en los pies tienen algún efecto en la calidad de mi vida Mis problemas en los pies tienen un efecto severo en la calidad de mi vida
Visual analogue scale (VAS) *How are your feet today? Please draw a line on the box below to the point of the scale that best indicate how good or bad your feet are today* Best imaginable 10 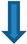 Worst imaginable 0	Escala visual analógica (EVA) *¿En qué estado de salud están hoy tus pies? Por favor marca el punto de la escala que mejor indique cómo están hoy de bien o mal tus pies siendo 0 el PEOR estado posible de salud del pie y 10 el MEJOR posible de salud del pie* Best imaginable 10 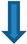 Worst imaginable 0

**Table 2:** Results of reliability, test–retest and systematic differences of the Spanish Podiatry Health Questionnaire (PHQ-S) according to each domain.

PHQ-S	Test *n* = 52			Retest *n* = 52			Reliability Test					
Domain	Mean ± SD (CI 95%) ^†^	Item–Total Correlation	α if Item Removed	Mean ± SD (CI 95%)^†^	Item–Total Correlation	α if Item Removed	ICC (IC 95%)	*p*-Value *	SEM	%SEM	MDC	%CV
Walking	1.71 ± 0.66 (1.52–1.89)	0.81 **	0.75	1.71 ± 0.66 (1.52–1.89)	0.83 **	0.76	0.93 (0.87–0.96)	1.000	0.00	0.00	0.00	0.00
Hygiene	1.21 ± 0.49 (1.07–1.35)	0.73 **	0.769	1.23 ± 0.50 (1.09–1.36)	0.72 **	0.79	0.93 (0.88–0.96)	0.317	0.00	0.30	0.01	1.15
Nail care	1.50 ± 0.64 (1.32–1.67)	0.67 **	0.79	1.53 ± 0.69 (1.34–1.73)	0.66 **	0.81	0.93 (0.88–0.96)	0.414	0.00	0.36	0.01	1.40
Foot pain	1.94 ± 0.69 (1.75–2.12)	0.81 **	0.757	1.96 ± 0.63 (1.79–2.13)	0.77 **	0.78	0.91 (0.85–0.95)	0.705	0.00	0.21	0.01	0.72
Worry and concern	1.73 ± 0.71 (1.53–1.93)	0.50 **	0.84	1.69 ± 0.61 (1.52–1.86)	0.58 **	0.83	0.90 (0.83–0.94)	0.480	0.00	0.50	0.02	1.65
Quality of life	1.55 ± 0.66 (1.37–1.74)	0.75 **	0.77	1.61 ± 0.69 (1.42–1.80)	0.78 **	0.78	0.871 (0.775–0.92)	0.366	0.01	0.96	0.04	2.68
Total	9.85 ± 2.7 (9.11–10.59)	N/A	N/A	9.87 ± 2.75 (9.13–10.61)	N/A	N/A	0.99 (0.96–0.98)	0.567	0.00	0.00	0.00	0.14
	Total Cronbach α test: 0.81			Total Cronbach α retest: 0.82								
VAS	6.31 ± 2.56 (5.60–7.03)	−0.50 **		6.34 ± 2.22 (5.72–6.96)	−0.39 **		0.86 (0.77–0.91)	0.834	0.03	0.54	0.09	1.46

Abbreviations: SD, standard deviation; CI 95%, confidence interval of 95%; CV, coefficient of variation; ICC, intraclass correlation coefficient; N/A, not applicable; * Wilcoxon signed-rank test; ** *p*-value < 0.001 (**in bold**); ^†^ values are expressed as lower–upper limits of the 95% confidence interval.

## Data Sharing Statement

The dataset supporting the conclusions of this article is available in the daniellopez@udc.gal in the Research, Health and Podiatry Unit. Department of Health Sciences. Faculty of Nursing and Podiatry. Universidade da Coruña, Spain.
